# Statistical analysis plan for a stepped-wedge cluster randomized clinical trial for the evaluation of the clinical impact of different telemedicine practices in intensive care units – TELESCOPE 2 trial

**DOI:** 10.62675/2965-2774.20260013

**Published:** 2026-10-23

**Authors:** Tiago Mendonça dos Santos, Renato Carneiro de Freitas Chaves, Bruna Gomes Barbeiro, Maura Cristina dos Santos, Thiago Domingos Corrêa, Alexandre Biasi Cavalcanti, Ary Serpa, Carlos Henrique Sartorato Pedrotti, Fernando Godinho Zampieri, Guilherme de Paula Pinto Schettino, Jorge Ibrain Figueira Salluh, Leandro Utino Taniguchi, Leonardo José Rolim Ferraz, Luciano Cesar Pontes Azevedo, Otávio Berwanger, Regis Goulart Rosa, Renata Albaladejo Morbeck, Rodrigo Santos Biondi, Suzana Margareth Lobo, Jessica Kasza, Adriano José Pereira, Otavio Ranzani

**Affiliations:** 1 Hospital Israelita Albert Einstein São Paulo SP Brazil Hospital Israelita Albert Einstein - São Paulo (SP), Brazil.; 2 Insper Instituto de Ensino e Pesquisa São Paulo SP Brazil Insper Instituto de Ensino e Pesquisa - São Paulo (SP), Brazil.; 3 Hospital Santa Luzia Luziânia GO Brazil Hospital Santa Luzia - Luziânia, GO, Brazil.; 4 Massachusetts Institute of Technology Cambridge MA United States Massachusetts Institute of Technology - Cambridge, MA, United States.; 5 Research Institute HCor São Paulo SP Brazil Research Institute, HCor-Hospital do Coração - São Paulo (SP), Brazil.; 6 Australian and New Zealand Intensive Care Research Centre Monash University Melbourne Australia Australian and New Zealand Intensive Care Research Centre, Monash University - Melbourne, Australia.; 7 Brazilian Research in Intensive Care Network São Paulo SP Brazil Brazilian Research in Intensive Care Network - São Paulo (SP), Brazil.; 8 Universidade Federal do Rio de Janeiro Rio de Janeiro RJ Brazil Universidade Federal do Rio de Janeiro - Rio de Janeiro (RJ), Brazil.; 9 Hospital das Clínicas Faculdade de Medicina Universidade de São Paulo São Paulo SP Brazil Hospital das Clínicas, Faculdade de Medicina, Universidade de São Paulo - São Paulo (SP), Brazil.; 10 Hospital Sírio-Libanês São Paulo SP Brazil Hospital Sírio-Libanês - São Paulo (SP), Brazil.; 11 Hospital Moinhos de Vento Porto Alegre RS Brazil Hospital Moinhos de Vento - Porto Alegre (RS), Brazil.; 12 Hospital Brasília Rede Américas Brasília DF Brazil Hospital Brasília - Unidade Lago Sul (Rede Américas) - Brasília (DF), Brazil.; 13 Hospital de Base de São José do Rio Preto Faculdade de Medicina de Ribeirão Preto São José do Rio Preto SP Brazil Hospital de Base de São José do Rio Preto, Faculdade de Medicina de Ribeirão Preto - São José do Rio Preto (SP), Brazil.; 14 School of Public Health and Preventive Medicine Monash University Melbourne Victoria Australia School of Public Health and Preventive Medicine, Monash University - Melbourne, Victoria, Australia.; 15 DataHealth Lab Institut de Recerca Sant Pau Barcelona Spain DataHealth Lab, Institut de Recerca Sant Pau (IR SANT PAU) - Barcelona, Spain.

**Keywords:** Telemedicine, Critical care, Intensive care units, Quality improvement, Clinical outcomes

## Abstract

**Background:**

The optimal model for delivering Tele-ICU is still to be determined. In the TELE-critical Care verSus usual Care on ICU Performance (TELESCOPE) trial, implementing medical-led daily multidisciplinary rounds did not show evidence of improved clinical outcomes. The TELESCOPE 2 trial was therefore designed to evaluate whether expanding this model to include a structured multidisciplinary care bundle, along with a formalized quality and safety management framework, would translate process improvements into measurable patient-centered outcome benefits.

**Objective:**

To evaluate whether a structured Tele-ICU intervention, comprising intensivist-led daily multidisciplinary rounds, a multidisciplinary care bundle (nursing, physical therapy, pharmacy), and a quality and safety management package, can reduce intensive care unit length of stay in Brazilian public intensive care units.

**Methods:**

TELESCOPE 2 is a multicenter, open-label, stepped-wedge cluster randomized trial including 25 intensive care units in Brazil from January 2024 to January 2026. Intensive care units are randomized to five sequences, receiving the intervention at staggered times. All adult patients admitted to participating units are eligible, except those admitted for non-medical reasons or those previously included in the study. The multifaceted telemedicine intervention comprises: i) daily multidisciplinary rounds led by board-certified intensivists; ii) coordinated care by a multidisciplinary team; and iii) a management strategy focused on quality improvement and patient safety. The primary outcome is intensive care unit length of stay. Secondary outcomes include clinical endpoints (mortality, ventilator-free days at 28 days, and ventilator-associated events), process-of-care indicators (mobilization, head-of-bed elevation, and sedation management), and unit-level organizational outcomes.

**Conclusion:**

We describe the statistical analysis plan for the TELESCOPE 2 trial, finalized prior to database lock. This pre-specified approach mitigates analysis bias and ensures the transparency and robustness of the reported results.

## INTRODUCTION

Tele-intensive care unit (ICU) is a technology-enabled model of care that allows intensivists to remotely support bedside teams. This approach has gained traction as a response to the uneven distribution of intensivists and limited access to critical care in underserved regions of Brazil, as in several other countries.^(1)^

Tele-ICU interventions, such as remote daily multidisciplinary rounds (DMRs), real-time clinical support, and performance monitoring, have been associated with improved patient care, including decreased ICU mortality, shorter hospital length of stay (LOS), and faster responses to alarms. Nevertheless, the best delivery model of Tele-ICU is uncertain.^(1)^

The two randomized clinical trials (ERIC and TELESCOPE-I trials)^(2,3)^ focused on ICU settings did not show direct improvements in clinical outcomes, although they observed improvements in certain care processes.^(2)^ In the ERIC trial, a telemedical quality improvement program significantly increased adherence to predefined performance metrics in acute ICU care in hospitals in the metropolitan area of Berlin.^(2)^In contrast, the TELESCOPE-I trial did not observe comparable improvements in process indicators, and telemedicine-based DMRs led by a board-certified intensivist did not reduce ICU LOS among critically ill adults.^(3)^

One of the potential reasons for the lack of success in showing benefit in clinical outcomes was the delivery model, i.e., intensivist-centered, without specifically accounting for the clinical multidisciplinary team and organizational factors. Tele-ICU is inherently a complex intervention, and both its delivery model and the range/skills of professionals involved are likely critical determinants of its effectiveness and its capacity to translate process improvements into meaningful patient-centered outcomes. While the feasibility and benefits of tele-ICU are well documented, evidence remains limited on the impact of different delivery models, such as integrating telemedicine with multidisciplinary care and quality management strategies.

The TELESCOPE-2 study aims to evaluate whether a structured telemedicine intervention, comprising DMRs led by an intensivist, a multidisciplinary care bundle (nursing, physical therapy, pharmacy), and a quality and safety management package, can reduce ICU LOS in Brazilian public ICUs.

## METHODS

### Study design

The TELESCOPE-2 study is an open-label, national, multicenter, stepped-wedge cluster randomized controlled trial. The unit of randomization is the ICU, as the intervention targets the entire multidisciplinary team. We will randomize 25 ICUs into five sequences (Figure 1). The Supplementary Material provides a complete list of participating centers and trial committee members.

**Figure 1:**
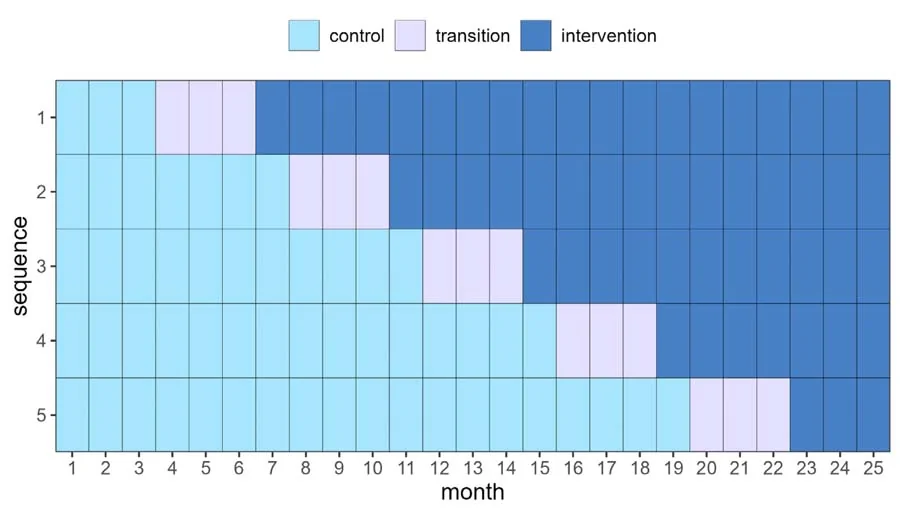
Schematic diagram illustrating the trial timeline.

Five intensive care units are randomized to each sequence.

Each sequence includes a control period without intervention, followed by staggered implementation of the intervention according to the assigned sequence. Given the complexity of the intervention package, we anticipate a delay in achieving the full effect of the intervention package. Therefore, all sequences include a transition period during which the intervention implementation will commence, but data collected during the transition period will be excluded from the primary analysis. All ICUs will eventually receive the full intervention package at different time points, enabling both within- and between-sequence comparisons.

### Randomization and allocation

Centers will be randomized into five sequences using a stratified approach. Randomization will use preliminary data from the first 2 months of the baseline period. The first baseline period will consist of 3 months; however, the third month will be designated for organizational preparation and disclosure of allocation for the first sequence.

The stratification will be based on two variables: geographic region and ICU LOS.

Region (Figure 2)

**Figure 2:**
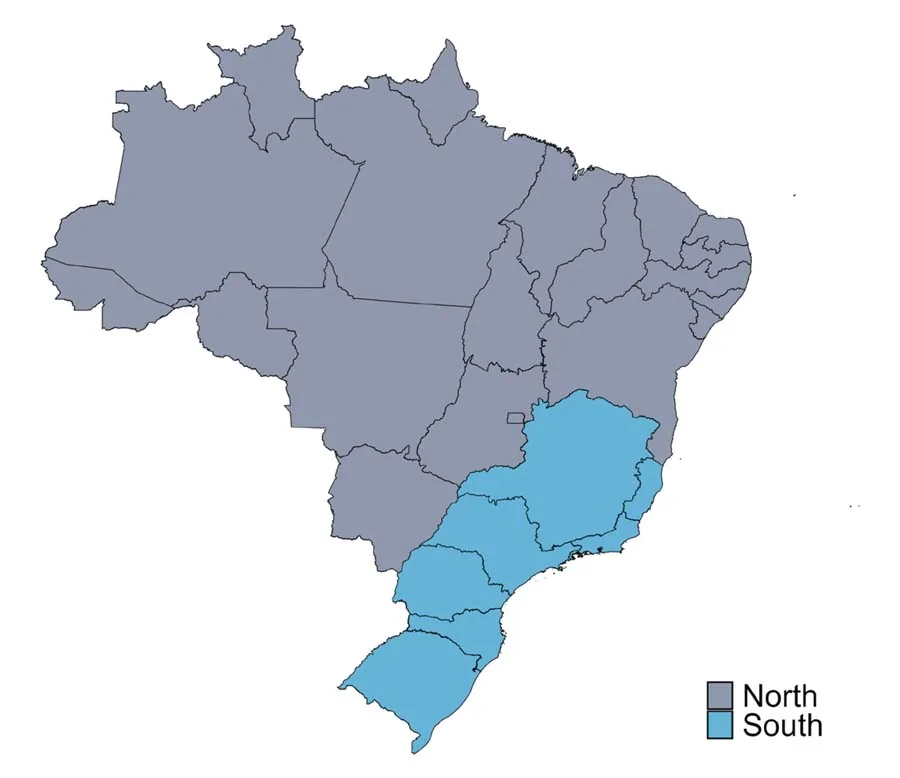
Regional grouping of Brazilian states for randomization purposes.

- South: centers located in the South and Southeast regions.
- North: centers located in the Central-West, North, and Northeast regions.

ICU LOS:

- Below: centers whose median ICU LOS is lower than the median for their respective region.
- Above: centers whose median ICU LOS is higher than the median for their respective region.

This stratification, generating four strata, was adopted to enhance balance across sequences, as geographic region in Brazil is associated with several structural and healthcare-related characteristics.^(3,4)^The stratum combining the South region and below-median LOS included seven centers, while the other three strata included six centers each.

All 25 ICUs will be randomized simultaneously using a computer-generated allocation list prepared by an independent statistician. The randomization will be constrained to a subset of sequences to ensure regional balance and approximate balance in baseline ICU LOS across sequences (and within strata), to account for the uneven number of centers by stratum. Specifically, sequences 1, 3, and 5 will be constructed to include one additional center from the South region, while sequences 2 and 4 will include one additional center from the North region. Table 1 details the exact distribution of centers across the four strata (region and ICU LOS) for each sequence. We will exclude candidate allocations that do not meet the region and baseline LOS constraints, and select one allocation uniformly at random from the eligible allocations. This constrained randomization will preserve overall balance while maintaining the probabilistic nature of the allocation.^(5)^

**Table 1:** Distribution of centers by sequence according to stratification variables (Region and intensive care unit length of stay)

Sequence	North/below median LOS	North/above median LOS	South/below median LOS	South/above median LOS	Total centers
1	1	1	2	1	5
2	1	2	1	1	5
3	1	1	1	2	5
4	2	1	1	1	5
5	1	1	2	1	5
Total	6	6	7	6	25

LOS - length of stay; ICU - intensive care unit.

Allocation concealment will be ensured by restricting access to the randomization sequence to the statistician, who will only have access to ICU identification codes and will be blinded to unit identities. The allocation list will then be provided to the study coordinator, who will disclose each center’s assignment 30 days before the transition phase to allow for organizational preparation.

The randomization will be performed using R (version 4.2.2).^(6)^ Primary analyses will adjust for region and baseline LOS category, thereby acknowledging the constrained randomization.

### Sample size calculation

We determined the required sample size by simulating a mixed-effects model for ICU LOS using one thousand replications per scenario and the Kenward-Roger correction.^(7)^ The model used the natural logarithm transformation of ICU LOS as the outcome to handle the right-skewed distribution, and included fixed effects for the intercept (2.079), a time trend (-0.012 i.e. a decrease in log-LOS of 0.012 unit per month), and the intervention effect (-0.148), corresponding to a 13.75% reduction from a baseline mean of 8 days (i.e., a reduction of 1.1 days). The random effects were characterized by: cluster-level variability following a normal distribution with a variance of 0.102; a period-within-cluster effect following a normal distribution with a variance of 0.019; and an individual-level residual error following a normal distribution with a variance of 1.090. These values yield an estimated intracluster correlation coefficient (ICC) of 0.10 and a cluster autocorrelation coefficient (CAC) of 0.84. These empirical estimates were derived from the TELESCOPE-I study.^(3)^ Based on these simulations, a total sample size between 18,750 and 25,000 patients is required to achieve at least 95% power at a two-sided significance level of 5%. This range reflects an expected monthly recruitment rate of 30 to 40 patients per ICU.

Because center withdrawal is a potential contingency in multicenter studies, we assessed the design’s robustness under two scenarios: removal of three centers from the first sequence and removal of three centers from the last sequence. Under a conservative scenario with 30 patients per ICU per period and removal of three centers from any sequence, power remains at least 93%.

### Interim analysis

Because our intervention incorporates the best available evidence for the care of critically ill patients in ICUs, and no inherent risks are anticipated in the trial’s execution, we did not plan interim analyses. As a result, we deemed a formal data monitoring committee unnecessary.

**Eligibility criteria:** the selected public and philanthropic ICUs were invited via electronic communication to participate in an interview to assess the inclusion and exclusion criteria, using an electronic feasibility assessment questionnaire. All patients admitted to the included ICUs will be eligible for inclusion in the study. The inclusion and exclusion criteria for both ICUs and eligible patients are detailed below.

**Inclusion criteria for ICUs:** public or philanthropic hospitals with 7 to 20 beds; availability of physicians and nurses 24 hours a day and a physiotherapist available for at least 18 hours a day.

**Exclusion criteria for ICUs:** (i) ICUs with structured multidisciplinary round, defined as meetings (DMRs) ≥ 3 times per week, during weekdays, conducted by a certified intensivist and documented in medical records with fixed visit length (> 5 minutes/patient), using some supporting tool (checklist or standard form), goal-oriented, based on established protocols, including all the patients admitted to the ICU; or (ii) ICUs with implemented monthly management of indicators (audit and feedback) with specific planning; or (iii) coronary or cardiac intensive care units, or other specialized units (e.g., neurological, or burn units); or (iv) step-down units or intermediate care units; or (v) ICUs without renal replacement therapy availability; or (vi) ICUs in which coordinators are board-certified intensivists and qualified as Masters of Business Administration (or an equivalent).

**Patient inclusion criteria:** adults (≥ 18 years old).

**Exclusion criteria for patients:** admissions for non-medical reasons (e.g., judicial) and previously included in the Telescope 2 study.

### Outcomes

The follow-up period to define all outcomes will be truncated at 90 days from ICU admission.

### Primary outcome

The primary outcome of this trial at the patient level is ICU LOS, defined as the time interval in hours between ICU admission and the time of ICU discharge (i.e., transfer to another care facility or hospital) or ICU death, as defined by the hospital’s system date and time. Date and time will be entered by the health care worker responsible for data collection. Intensive care unit LOS will be derived in 24-hour periods with decimal places, as recommended.^(8)^

### Secondary outcomes

- ICU mortality, defined as death by any cause during the index ICU admission.^(3)^
- In-hospital mortality, defined as death by any cause during the index hospital admission.^(3)^
- Ventilator-free days during the first 28 days, defined as the number of days alive and free from mechanical ventilation for at least 24 consecutive hours from successful weaning to day 28. Patients who die before weaning will be deemed to have zero ventilator-free days, and patients discharged from the hospital before 28 days will be considered alive and free from mechanical ventilation at 28 days.^(3)^
- ICU readmission within 48 hours.^(3,9)^
- Early reintubation (< 48 hours after elective extubation).^(3)^
- Ventilator-associated events: defined as either an increase in the daily minimum positive end-expiratory pressure (PEEP) of ≥ 3cmH_2_O sustained for at least two consecutive calendar days following a period of at least 2 days of stable or decreasing PEEP, or an increase in the fraction of inspired oxygen (FiO_2_) by ≥ 20 percentage points sustained for at least two consecutive days after a minimum of 2 days of stable or decreasing FiO_2_ levels.^(10)^
- Accidental extubation rate.^(3)^

### Process-of-care and quality indicators

- Patient mobilization density in the ICU: defined as the number of days the patient undergoes any form of mobilization, including passive movement of upper or lower limbs in bed, sitting on the edge of the bed or in a chair, or walking.^(2,11,12)^
- Adherence to maintaining the head-of-bed elevation (30°- 45°).^(3,11)^
- Adequate prevention of venous thromboembolism.^(11,13)^
- Rate of patient-days under adequate sedation (defined by Richmond Agitation and Sedation Scale [RASS] = -3 to +1).^(11,14)^
- Rate of patient-days with oral or enteral nutrition.^(2,11)^
- Rate of patients with adequate glycemic control (defined as blood glucose between 70 to 180mg/dL).^(3,11,13)^
- Rate of patient-days within normoxemia (defined as peripheral oxygen saturation = 92 to 96%).^(3)^
- Rate of central venous catheter use.^(3,11)^
- Central venous catheter dwell time.^(3,11)^
- Rate of indwelling urinary catheter use.^(3,11)^
- Indwelling urinary catheter dwell time.^(3,11)^

### Unit-level organizational outcomes

–ICU classification by standard resource use (SRU) and standardized mortality rate (SMR): defined by SRU and SMR.^(13)^ Standard resource use reflects the observed-to-expected rate of resource utilization, estimated as ICU LOS for surviving patients and adjusted for the patient’s severity of illness. Standardized mortality rate reflects the observed-to-expected rate of hospital deaths, according to the Simplified Acute Physiology Score (SAPS 3). The profiles combine SMR (above or below median) and SRU (above or below median). Each unit will be assigned to one of four groups: “most efficient” (SMR and SRU below median); “least efficient” (SMR, SRU above median); “overachieving” (low SMR, high SRU), “underachieving” (high SMR, low SRU).^(15)^

### Statistical principles

All statistical tests will be two-sided, and all analyses will use a significance level of 5%. Primary analyses will follow the intention-to-treat principle, with patients analyzed according to their randomized ICU assignment, regardless of intervention exposure (i.e., ICUs will be considered to have switched to the intervention at their scheduled time). We will report findings following the standards established by the CONSORT extension for stepped-wedge trials.^(16)^

Analyses will be conducted in R (version 4.3.3 or later; Vienna, Austria). Mixed-effects models (described below) will primarily be fitted with the lme4 package.^(17)^ For continuous outcomes, due to the reduced number of clusters, the Kenward-Roger small-sample correction will be applied. Prespecified secondary outcomes and subgroup analyses will not be adjusted for multiple comparisons and will be considered exploratory.

Multiple imputation will be performed if missing data on core variables exceed 10%, following standard procedures using chained equations with the mice package.^(18)^

### Primary outcome analysis

We will analyze the primary outcome, ICU LOS, using a linear mixed-effects model on the natural logarithm of ICU LOS (days). This transformation addresses the typically right-skewed distribution of LOS. The model will include both fixed and random effects to account for the hierarchical structure of the stepped-wedge design, the stratified randomization variables, and the patient-level SAPS 3 score.

Formally, the model is defined as:


$$\begin{array}{cc} & \gamma_{kti}=\beta_{0}+\beta_{1}X_{1kt}+\beta_{2}X_{2k}+\beta_{3}X_{3k}+\beta_{4t}T_{t}+\beta_{5}{\mathrm{SAPS}}_{kti}+\alpha_{k}\\ & +\gamma_{tk}+\varepsilon_{kti} \end{array}$$

Where:

- *ɣ*_*kti*_: log- transformed ICU LOS for patient *i* at time period *t* in cluster *k*.
- *β*_0_: fixed intercept (expected log-LOS at baseline).
- *β*_1_: fixed effect for the intervention.
- *X*_1*kt*_: intervention indicator for cluster *k* at time *t*, defined as one for intervention and zero for control.
- *β*_2_: fixed effect for the stratification variable of Brazilian region.
- *X*_2*k*_: regional location for cluster k, defined as zero for North region centers and one for South region centers.
- *β*_3_: fixed effect for the stratification variable of ICU LOS category.
- *X*_3*k*_: baseline ICU LOS category for cluster *k,* defined as 0 for below-median and 1 for above-median within region.
- *β*_4*t*_: fixed effect for calendar time.
- *T*_*t*_: factor for month.
- *β*_5_: fixed effect for SAPS 3.
- *SAPS3*_*kti*_: SAPS 3 for patient *i* in period t and cluster k.
- *α*_*k*_ ~ *N* (0,*σ*_*α*_^2^): random intercept for cluster *k* (ICU), capturing between-cluster variability.
- *ɣ*_*tk*_ ~ *N* (0,*σ*_*γ*_^2^): random effect for time within cluster, accounting for within-cluster temporal correlation.
- *ε*_*kti*_ ~ *N* (0,*σ*_*ε*_^2^): individual-level residual error.

This model accounts for clustering and repeated measures within each ICU and allows estimation of the intervention effect while adjusting for temporal trends and between-cluster variability.

### Secondary, process-of-care, and unit-level organizational outcomes analysis

We will analyze continuous secondary and exploratory outcomes using the same mixed-effects modeling framework as the primary outcome, including the same fixed and random effects. Count and rate outcomes will be analyzed using generalized linear mixed-effects models with Poisson or negative binomial distributions, as appropriate, incorporating the same fixed- and random-effects structure as the primary model.

We will analyze binary outcomes using generalized linear mixed-effects models with a logit link function, incorporating the same set of independent variables as in the primary model. These models will account for clustering at the ICU level and repeated measures over time.

For categorical outcomes with more than two categories, we will fit multinomial logistic mixed-effects models with ICU random intercepts at the cluster (ICU) level. We will use the same covariate structure as in the primary outcome model. These models will allow for estimation of category-specific odds ratios while accounting for hierarchical data structure.

### Sensitivity analysis for the primary outcome

We will assess the robustness of the primary analysis to alternative modeling choices and design assumptions as follows:

- Covariate adjustments: we will repeat the primary analysis additionally adjusting for the following covariates: type of ICU admission (three categories: medical, elective surgical, unplanned surgical), invasive mechanical ventilation at ICU admission (two categories: yes, no), number of ICU beds at baseline (continuous term), and ICU performance at baseline (four categories: most efficient, least efficient, overachieving, underachieving). For baseline covariates, we will define baseline using data from the first 2 months of the study.
- Alternative correlation structures: we will assess the robustness of our results to different assumptions regarding the within-cluster correlation. This will include fitting a model that considers the cluster as the only random effect and allows a discrete-time decay model to account for the potential weakening of correlation over time.
- Readiness for discharge: to minimize bias from non-clinical delays (e.g., weekends or ward-bed availability), we will redefine ICU LOS as time from ICU admission to the calendar day on which the patient first met readiness-for-discharge criteria.^(3)^
- Transition window: we will also analyze the transition period as part of the intervention to account for potential effects during this phase.
- Competing risk: because the primary endpoint, ICU LOS, combines two processes (discharge alive and ICU death), death functions as a competing event for discharge. To assess robustness, we will reanalyze time to ICU discharge alive within a competing-risk framework that treats death as a competing event. We will present cause-specific hazard ratio estimates and cumulative incidence for discharge alive.^(19)^
- Early mortality exclusion: we will repeat the primary analysis after excluding patients who died within the first 24 hours of ICU admission. Because the intervention has little to no opportunity to affect outcomes among patients who are critically ill on arrival and die very early, this analysis assesses whether such early deaths dilute or distort the estimated intervention effect.

### Time-dynamic effects

We will include an interaction between intervention and time since implementation within ICU to assess potential ramp-up or waning of effect over time. For this exploratory analysis, we will also include the transition period for each sequence. Following the exposure time indicator framework, we will report treatment effects as separate point estimates for each month since implementation.^(20)^

### Subgroup analysis

We will conduct prespecified subgroup analyses by adding interaction terms between the intervention and each subgroup variable in the primary outcome model. The following subgroups will be explored:

- Type of admission (medical *versus* surgical).
- Telemedicine experience (centers with *versus* without prior telemedicine use).
- SAPS3 score (categorized by tertiles).
- Mechanical ventilation status at admission (invasive *versus* non-invasive *versus* none).
- ICU performance at baseline (four categories: most efficient, least efficient, overachieving, underachieving). We will make each classification based on the first two months of the initial baseline period.

These analyses will be exploratory. We will interpret estimates of interaction effects with caution, without adjustment for multiple comparisons.

### Handling of timing in stepped-wedge design

In this stepped-wedge cluster randomized trial, the timing of intervention rollout across ICUs is a key component of the analytical structure. Each ICU contributes data during control and intervention phases, separated by a transition period during which the intervention is being implemented but is not yet assumed to have reached full effect.

For the primary analysis, we will include only patients admitted during the defined control and intervention periods. We will exclude patients admitted during the transition period, as this phase is expected to involve partial uptake and variability in adherence to the intervention protocols.

We will model calendar time as a fixed effect in all primary and secondary analyses to adjust for potential secular trends and other time-related confounders. This approach helps isolate the intervention effect from temporal changes unrelated to the study.

By excluding transition periods from the main analysis and explicitly accounting for time in the statistical model, we aim to minimize bias due to gradual implementation and better estimate the true effect of the intervention.

### Brazilian healthcare system

The Brazilian healthcare system comprises three main sectors: public, philanthropic, and private.^(21)^The public healthcare system is a universal health system established by the Constitution, which guarantees healthcare as a right of all citizens and a duty of the State. The public healthcare system is financed through federal, state, and municipal taxes. It provides comprehensive care, ranging from primary care and vaccination programs to high-complexity services such as ICU treatment, free of charge at the point of delivery. The philanthropic sector may provide care to patients funded by the public system, as well as to those covered by private insurance or paying out of pocket. Patients admitted under the public system in these institutions receive treatment free of charge. In parallel, a supplementary private sector, funded through private insurance plans or direct out-of-pocket payments, serves a portion of the population and frequently overlaps with public service provision. Although the public healthcare system has achieved substantial public health advances, including expanded access and improved health indicators, it continues to face challenges related to regional disparities, funding limitations, workforce distribution, and access to specialized and high-complexity care services. In the TELESCOPE-2 trial, ICUs were eligible only if they exclusively treated patients admitted under the public healthcare system.
